# Clinician Decisions After Notification of Elevated Blood Pressure Measurements From Patients in a Remote Monitoring Program

**DOI:** 10.1001/jamanetworkopen.2021.43590

**Published:** 2022-01-14

**Authors:** Natalie S. Lee, Rebecca Anastos-Wallen, Krisda H. Chaiyachati, Catherine Reitz, David A. Asch, Shivan J. Mehta

**Affiliations:** 1 Division of General Internal Medicine, The Ohio State University Wexner Medical Center, Columbus; 2 Department of Medicine, Perelman School of Medicine, University of Pennsylvania, Philadelphia; 3 Leonard Davis Institute of Health Economics, University of Pennsylvania, Philadelphia; 4 Center for Healthcare Innovation, University of Pennsylvania, Philadelphia

## Abstract

**Importance:**

Guidelines recommend using telehealth for hypertension management, but insufficient evidence is available to guide strategies for incorporating telehealth data into clinical practice.

**Objective:**

To describe how primary care teams responded to elevated remote blood pressure (BP) alerts in the electronic health record (EHR) in a randomized clinical trial of BP telemonitoring conducted in routine practice settings.

**Design, Setting, and Participants:**

This retrospective cohort study reviewed EHR documentation from May 8, 2018, to August 9, 2019, in a single urban academic family practice site. Primary care teams comprising 28 attending physicians and nurse practitioners, residents, and nurses cared for 162 patients in a text-based clinical trial of remote BP monitoring remote BP monitoring. Data were analyzed from October 21, 2019, to April 30, 2021.

**Exposures:**

Clinicians received a direct message in their EHR inbox when patients submitted at least 3 elevated BP readings.

**Main Outcomes and Measures:**

Categories and frequencies of clinician action, created via review of EHR-documented clinician responses to EHR alerts by 2 physicians.

**Results:**

Patients in this study (n = 162) were predominantly female (111 [68.5%]) and Black or African American (146 [90.1%]), whereas attending physicians (n = 21) were predominantly female (13 [61.9%]) and non-Hispanic White (19 [90.5%]) with a mean (SD) age of 51.6 (11.1) years. Five hundred fifty-two alerts fell into 12 categories of clinical actions. Clinicians acted on 343 alerts (62.1%). Common remote activities were to reconcile medications and assess adherence (120 of 552 alerts [21.7%]) and verify BP measurement technique (65 of 552 alerts [11.8%]). Clinicians also commonly requested appointments (120 of 552 alerts [21.7%]) and/or saw the patient in a subsequent office visit (114 of 552 alerts [20.7%]). Ninety-six alerts (17.4%) resulted in medication changes; half of these changes were remote (48 of 96 [50.0%]), and the other half were visit-based. For 209 of 552 alerts (37.9%), no changes were made to the care plan, typically without documenting clinical rationale (196 of 209 instances [93.8%]). Exploratory EHR review was used to infer potential clinical rationale for 106 (54.1%) of such cases, but there was insufficient information for the remaining 90 (45.9%).

**Conclusions and Relevance:**

These findings suggest that EHR alerts for elevated BP during remote monitoring were effective in prompting a mix of remote and office-based management. It was also common for the plan of care to remain unchanged, possibly suggesting need for more refined alerts and improved clinician support.

## Key Points

**Question:**

How do clinical teams respond to electronic health record (EHR) notification of elevated blood pressure (BP) for hypertension telemonitoring?

**Findings:**

In this cohort study using data from a randomized clinical trial of remote BP monitoring from 162 patients, clinicians acted on 343 (62.1%) of 552 EHR alerts for persistently elevated BP home readings, with a mix of remote and office-based management. There were no changes to the care plan for the remaining 209 alerts (37.9%), often because alerts did not account for other pertinent information such as office-based readings and upcoming appointments.

**Meaning:**

Electronic health record alerts may be an effective strategy for transmitting telehealth data to clinicians, with some limitations in clinical utility and impact.

## Teaser

This cohort study uses data from a randomized clinical trial to assess the range of clinician responses to elevated blood pressure alerts via the electronic health record for patients receiving hypertension telemonitoring.

## Introduction

Although hypertension is the most common attributable risk factor for cardiovascular and cerebrovascular disease,^1,2^ awareness and control remain limited in the US.^3^ Remote care will play an increasing role in hypertension management. Home blood pressure (BP) monitoring is currently recommended to guide management,^4^ and transmission of those data to clinicians likely provides added benefit.^5,6^ Guidelines recommend telehealth strategies for managing hypertension,^7^ and new codes from the Centers for Medicare & Medicaid Services reimburse remote monitoring of physiological parameters.^8^

Despite these trends, little is known about how telehealth data should be integrated into clinical care. Technologies and platforms to support BP telemonitoring are increasingly available, from websites to email or smartphone applications.^9,10^ For most health systems, the electronic health record (EHR) is the hub of clinical activity and therefore a logical place for telehealth data integration. Alerts are routinely integrated into contemporary EHRs to support clinical care, such as medication flags or best-practice alerts.^11,12,13,14^ In practice, however, there is also increasing concern that clinicians are overloaded with EHR alerts and often dismiss them.^15,16,17^

Additional research is therefore needed on the fate of EHR clinician alerts for hypertension telemonitoring. The goal of this study was to describe the range of clinician responses to elevated BP alerts via the EHR in a clinical trial of remote BP monitoring.

## Methods

### Study Context

This cohort study was a secondary analysis of data from a 16-week randomized clinical trial conducted from May 8, 2018, to August 9, 2019, at an academic family medicine practice site within the University of Pennsylvania Health Systems in Philadelphia.^18^ The practice consists of 28 family physician and nurse practitioner primary care clinicians, plus a team of nurses and rotating residents. During the time of the study, 21 attending physicians practiced at the site. Their demographic information was obtained from the University of Pennsylvania School of Medicine faculty database to describe clinician characteristics. Demographic information for nurse practitioners, nurses, and residents at the practice was not available. This secondary analysis was approved under expedited review as an amendment to the underlying study by the University of Pennsylvania Institutional Review Board. Patients provided verbal informed consent to participate in the underlying study. Clinicians provided usual care and did not consent. Reporting followed the Strengthening the Reporting of Observational Studies in Epidemiology (STROBE) guideline for observational cohort studies.

The underlying study was a 3-group clinical trial, with the intervention integrated into routine clinical care. The 2 primary purposes of the underlying study were (1) to assess the effects of remote BP monitoring and medication adherence via text message, with feedback submitted to both patients (via text) and clinicians (via EHR) if readings were not at target, and (2) to compare the impact of providing feedback via text to a social support partner identified by the patient. Thus, the 3 patient groups were (1) a control group in which patients received usual care, with prestudy and poststudy BP measurements being the only study-related activity; (2) a remote monitoring group in which patients self-reported BP and medication adherence via text message, with clinicians alerted via EHR when readings were uncontrolled; and (3) a remote monitoring plus social support group identical to group 2, with the addition of a social support partner who also received text message alerts when readings were uncontrolled. Eligible patients were identified in the EHR if they were adults aged 18 to 75 years with a diagnosis of hypertension (*International Statistical Classification of Diseases and Related Health Problems, Tenth Revision*, code I10); were prescribed at least 1 medication for hypertension; and had at least 2 visits to the practice within the past 24 months with uncontrolled BP readings, including at the last visit (systolic BP of ≥150 mm Hg and/or diastolic BP of ≥90 mm Hg; or systolic BP of ≥140 mm Hg and/or diastolic BP of ≥90 mm Hg for adults aged 21-59 years or those with chronic kidney disease or diabetes, according to Eighth Joint National Committee guidelines^19^). Race and ethnicity data were abstracted from the EHR and included as part of the sociodemographic description of the cohort. One hundred ninety-eight patients were randomized to the mobile text message intervention, with or without performance reports to a social support partner. Three times per week, patients were prompted via text to respond with their home BP readings, and once a week they were prompted to respond via text with the week’s medication adherence. Study coordinators (including C.R.) alerted clinicians via a documented message in the EHR only when (1) at least 3 of the last 10 BP readings were above target or (2) a single reading of systolic BP was 180 mm Hg or higher and/or diastolic BP was 110 mm Hg or higher. The notifications included at least 5 and as many as 10 of the most recent BP readings in chronological order, including any normal readings between abnormal readings, to provide more clinical context to the clinicians. An example message of the notification sent to clinicians is provided in the eMethods in the Supplement. Clinicians did not otherwise receive EHR alerts for normal BP readings. Electronic health record alerts were submitted directly to the 27 primary care clinicians in the first half of the study. In the second half of the study, based on primary care clinician feedback, escalations were instead sent to a nurse pool. Because this trial was integrated into routine care, clinicians were not provided explicit instructions regarding response to BP alerts.

The underlying study thus allowed the observation of real-world clinician responses to EHR alerts for abnormal patient-reported BP measurements. Of the 198 patients in both intervention groups, 162 met criteria for at least 1 escalation reported to the clinician during the study. The remaining 36 either did not meet criteria for escalation (n = 32) or were enrolled but did not submit any BP readings (n = 4).

### Review Process and Interreviewer Agreement

Two physicians (N.S.L. and R.A.-W.) reviewed the EHR records for the 162 patients who met escalation criteria. All alerts recorded in the EHR for these patients were reviewed, as well as the EHR documentation by the clinical team around the time of each alert for additional clinical context. The first 100 of the 162 patient records (61.7%) were reviewed collaboratively to develop and define categories of actions taken by clinicians. Once a codebook was finalized, the remaining patient records were reviewed independently to assess interreviewer agreement. We calculated Cohen κ correlation coefficients for 3 sets of observations. First, we measured interreviewer agreement about whether action was taken by the clinician. The κ coefficient was 0.96 (95% CI [0.92-0.99]), suggesting almost perfect agreement.^20,21^ Second, we examined the subset of alerts for which clinician action was taken, and we measured the interrater agreement for categorizing the specific type of action taken. The measured κ coefficient was 0.85 (95% CI [0.81-0.88]), again suggesting almost perfect agreement. Finally, we examined the other subset of alerts for which no changes were made and assessed interrater agreement classifying clinicians’ documented reasons for unchanged plan of care. The κ coefficient was 0.45 (95% CI [0.33-0.57]), suggesting moderate interrater agreement. After independent review, all discrepant ratings for the 3 sets of observations were resolved between the 2 reviewers through discussion.

### Further Exploratory Review and Statistical Analysis

During the review process, the 2 physician reviewers additionally explored the potential clinical reasons no changes were made, when clinical reasoning was not otherwise documented by the receiving clinician. Assessments were binary—clinical rationale identified vs not clearly identified—based on review of available EHR documentation and BP values. Assessments were not explicitly reconciled, but where they were discrepant, we defaulted to the reviewer who identified clinical rationale. The κ coefficient assessing interrater agreement for whether there was identifiable clinical rationale was 0.78 (95% CI [0.59-0.97]), suggesting substantial agreement. Reviewers also noted their interpretation of the specific reason for unchanged care plan, but there was no formal codebook defining subcategories of identifiable clinical rationale. Data were analyzed from October 21, 2019, to April 30, 2021. 

## Results

The 162 patients included in this analysis were predominantly female (111 [68.5%] women vs 51 men [31.5%]), with a mean (SD) age of 51.6 (11.1) years. Most patients were Black or African American (146 [90.1%] vs 8 [4.9%] non-Hispanic White and 8 [4.9%] all other/unknown), and privately insured (80 [49.4%] vs 41 [25.3%] Medicaid, 38 [23.5%] Medicare, and 3 [1.9%] uninsured/unknown). Across the 162 patients, 552 alerts were generated to clinicians. Twenty-one attending physicians were involved in the receipt of BP alerts. They were predominantly female (13 [61.9%] women vs 8 [38.1%] men). In terms of race and ethnicity, 1 physician was Hispanic (4.8%), 1 was non-Hispanic other (4.8%), and 19 were non-Hispanic White (90.5%).

**Figure 1.**
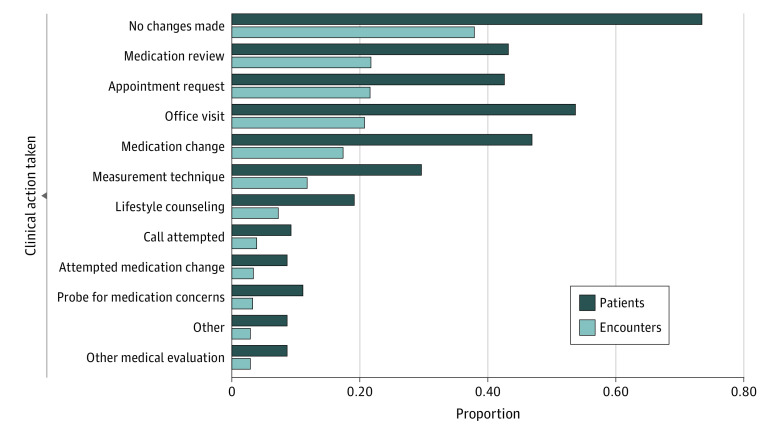
Frequency of Clinician Actions in Response to Elevated Blood Pressure (BP) Alerts by Encounter and Patient

**Figure 2.**
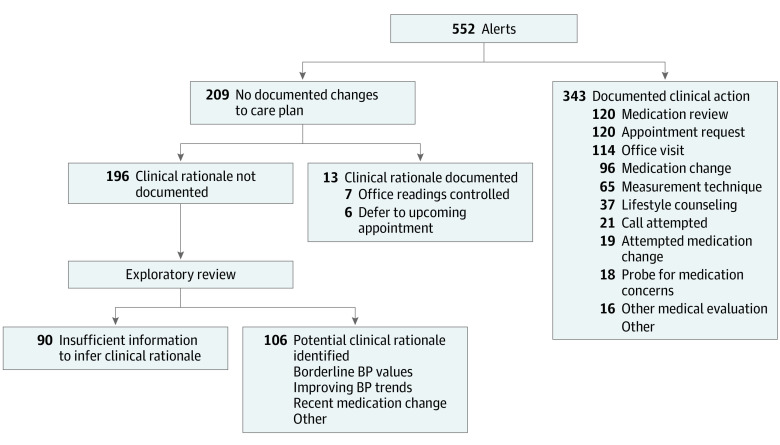
Flowchart of Clinician Responses to Elevated Blood Pressure (BP) Alerts A total of 162 patients were included in the study population.

The 552 alerts among 162 patients fell into 12 categories of clinical actions (Table and Figure 1), including inaction. Clinicians acted on 343 alerts (62.1%) (Figure 2). When action was taken, the mean (SD) time to clinician action, defined as the time of clinician documentation, was 3.2 (5.6) days.

**Table.** Categories of Actions Taken by Clinicians in Response to Elevated BP Readings Obtained Remotely

Category	Description	Alerts, No. (%) (n = 552)
Unchanged management	No action was documented in response to BP elevation.	209 (37.9)
Medication review	Antihypertensive medication list reviewed, and adherence assessed.	120 (21.7)
Appointment request	Patient instructed to schedule a follow-up appointment.	120 (21.7)
Office visit	A visit occurs in which BP management is addressed.	114 (20.7)
Medication change	Antihypertensive medications were added or adjusted.	96 (17.4)
Measurement technique	Accurate technique for obtaining BP measurement was reviewed.	65 (11.8)
Lifestyle counseling	Counseled on lifestyle modification, including smoking, diet, exercise, and managing anxiety.	37 (6.7)
Call attempted	Clinician initiated call but could not reach patient.	21 (3.8)
Attempted medication change	Clinician broaches medication change but patient declines or does not respond to outreach.	19 (3.4)
Probe for medication concerns	Clinician inquires about medication concerns (eg, adverse effects).	18 (3.3)
Other medical evaluation	Medical workup initiated, including secondary causes of hypertension, laboratory workup, evaluation of symptoms related to hypertension, etc.	16 (2.9)
Other	Action not otherwise captured in the other categories.	16 (2.9)

Abbreviation: BP, blood pressure.

Common actions involved both remote and office-based activities. Common remote activities were to reconcile medications and assess adherence (120 of 552 alerts [21.7%]) and verify BP measurement technique (65 of 552 [11.8%]). Other remote activities such as lifestyle counseling, assessment of patient concerns, or other medical evaluations were less common. Clinicians commonly requested appointments (120 of 552 alerts [21.7%]) and/or saw the patient in a subsequent office visit (114 of 552 [20.7%]). Medications were changed in response to 96 alerts (17.4%), with almost half of all study participants having their medications adjusted during the study (76 of 162 [46.9%]). Half of all occurrences of medication adjustments (48 of 96 [50.0%]) were associated with an office visit, while the other half occurred remotely.

Infrequently (19 of 552 alerts [3.4%]), clinicians attempted to adjust medication remotely, but the patient refused or did not reply. Actions that were categorized as other included education about the consequence of hypertension, assessment of medication affordability, referral to a specialist, and addressing acute pain as a cause of BP elevation (16 of 552 alerts [2.9%]).

No changes were made in response to 209 alerts (37.9%), affecting 119 patients (73.5%) at least once and 57 patients (35.2%) twice or more. For 13 of 209 such instances (6.2%), clinicians’ documented reasons were that BP readings had been controlled at a recent office visit (7 of 209 [3.3%]) or that management could be deferred to an upcoming appointment (6 of 209 [2.9%]). In most instances, however, there was no documentation of underlying clinical rationale (196 of 209 instances [93.8%]). In such cases, 130 of 196 alerts (66.3%) lacked any clinician documentation in response, whereas 66 of 196 (33.7%) were accompanied by clinician documentation merely acknowledging the alert, without further comment on clinical reasoning or circumstances.

### Exploration of Inaction Without Documented Clinical Rationale

The 2 physician reviewers examined the EHR documentation and BP trends that accompanied the 196 alerts for which care plans remained unchanged without documented clinical rationale. The reviewers inferred clinical reasoning for 106 of such instances (54.1%). These justifications included BP values being close to target, a downward trend in readings suggesting improvement, and a recent medication change with expected BP improvement. A comprehensive list of unique potential reasons no changes were made, as inferred by exploratory physician review, is provided in the eResults in the Supplement. For the remaining 90 unchanged care plans without documented clinical rationale (45.9%), insufficient information was available to infer clinical rationale (51 of 162 patients [31.5%]).

## Discussion

Although telemonitoring initiatives for chronic conditions such as hypertension are growing in use, the evidence needed to guide where and how telehealth information should be transmitted is insufficient. The EHR is the most intuitive place for storing and transmitting this information to clinicians, but little is known about how clinicians respond to it. In this secondary analysis of data from a randomized clinical trial, we evaluated clinician responses to persistently elevated home BP alerts transmitted through the EHR.

No protocols outlined how clinicians should interact with BP alerts in the underlying clinical trial. In that context, clinicians responded with clinical action to 62.1% of escalations. This finding is in line with previously reported rates of physician engagement with telemedicine BP reports of 49.5% to 85%.^22,23^ Almost half of all patients in the study (46.9%) had their medications adjusted. Although we did not have a usual care comparison group, estimates of medication adjustment rates for uncontrolled hypertension range from as low as 7% to roughly 17%.^24,25,26^ Prior studies also support more frequent medication therapy intensification with telemonitoring.^6,27^ Not all medication intensification is equal (eg, stepwise dose escalation vs fixed-dose combination pills or addition of new medication classes^28,29,30^), and our study did not examine differences in medication change that occurred remotely vs in person. Nevertheless, our observations suggest that overall, EHR alerts for persistently elevated home BP readings were reasonably effective in prompting a clinical response.

Notably, remote transmission of hypertension data to the clinician did not necessarily lead to remote hypertension management. Sometimes it did, because half of all medication adjustments in the study occurred remotely. More commonly, however, remote activities were limited in scope and primarily related to review of medications, adherence, and BP measurement technique. Much of the time, clinicians requested an appointment and/or saw the patient in the office for management, with more than half of all patients attending an in-person visit in response to a remote BP escalation. Data from older studies and other national health systems have not typically demonstrated an increase in office visits associated with telemedicine,^23,31,32,33,34,35^ but our findings are consistent with clinical experience—that remote BP management often requires more time, effort, and patient interaction than is conventionally available to the clinician outside the office visit. This may be why other successful programs often delegate remote BP management responsibilities across multidisciplinary team members, such as pharmacists or nonclinician navigators.^36,37,38^

The observed tendencies toward physical visit–based hypertension management also likely reflect a reimbursement schema that has not typically rewarded remote care activities. Post–COVID-19 policy changes and new Centers for Medicare & Medicaid Services reimbursement codes that account for non–face-to-face care management services may shift practice patterns, particularly the use of telemedicine instead of office visits.^39^ On the other hand, our observations may better reflect what practice patterns may be observed should existing pandemic-related regulatory waivers for telehealth expire.

This study provides insight into ways in which health systems might support clinicians as telehealth data are integrated into clinical practice. Common efforts to address clinical uncertainty, such as medication reconciliation, adherence assessments, and review of BP measurement techniques, could be protocolized or even automated before clinician review.^40^ Health systems might also anticipate a need for frequent clinician-patient engagement, whether they occur in an office or virtually.^41^

For 37.9% of all alerts, clinicians made no changes to the care plan. Inferred or explicit clinical reasons for no change included improving BP trends or anticipated improvement because of recently implemented medication changes. More sophisticated alerts that move beyond simple thresholds are likely essential, because low-utility EHR alerts are a growing and well-acknowledged problem leading to burden, fatigue, desensitization, and burnout.^17,42,43,44^ Our observations suggest that, at a minimum, future EHR alerts might account for clinically meaningful BP trends or recent management changes. Machine learning models could also be used to improve clinical alerts.^45,46^

For the 37.9% of alerts for which no changes were made, there was insufficient information to infer the rationale, which might have included any number of circumstances unique to the patient and not immediately obvious in the EHR. Further research is needed to understand the factors that facilitate or impede clinician response to remotely obtained information. Indeed, BP elevations observed in the office are often left unaddressed,^3,47^ suggesting that the effects we have observed may not reflect the processes of remote care so much as more general processes of, or barriers to, hypertension management.

### Strengths and Limitations

This study has important strengths. Few studies have described in detail the specific actions clinicians take for remote BP management. Team-based care is recommended for hypertension management,^7,48^ and our observations of clinician actions were for clinical teams of nurses and primary care clinicians; this is probably the most common team structure for other health systems looking to integrate telehealth data. The intervention was conducted under usual care conditions of a large urban primary care practice. An added strength is that our raters were physicians with clinical understanding of EHR documentation. All EHRs were reviewed by both physician reviewers, and all discrepant observations for categories of clinical action were eventually resolved through discussion.

This study also has several limitations. The findings reflect the practice of a group of academic providers at a family medicine site in Philadelphia. This study was completed before the COVID-19 pandemic began and therefore may not reflect postpandemic practice patterns, although it may reflect practice patterns should pandemic-related regulatory waivers expire. Study participants represented a small proportion of each clinician’s panel, so our observations may not apply to a scenario wherein a clinician was receiving alerts for an entire hypertensive panel. Moreover, all determinations were based on EHR documentation, and undocumented clinician actions were not observable. However, clinicians typically document patient care activities for the purposes of billing or communication with other clinicians, making it unlikely that we missed important patient-clinician interactions. Another consideration is that clinicians might have changed their behavior in response to being monitored, which could have biased our findings toward observing more clinician actions. However, because the study participants represented only a small portion of each clinician’s panel, we find it unlikely that observer bias contributed substantially to our results. Furthermore, there were no explicit criteria for assessing the appropriateness of clinician responses. Finally, we could not verify that a clinician had indeed viewed an elevated BP alert, and it is possible that the alert was not read. However, we assumed they likely had because alerts were sent as messages to the provider’s EHR inbox, which is typically a cornerstone of the clinician’s workflow.

## Conclusions

In total, 62.1% of EHR alerts for elevated telemonitored BP readings prompted clinical action, with both remote and office-based management of telehealth data. This finding suggests that the EHR may be an appropriate place to transmit abnormal telemonitored BP values to clinicians. However, alerts based on simple thresholds may lack clinical utility, because clinical action was often not necessary for the alerts generated. Other times, inaction was not justified, and further research is needed to understand how best to integrate telehealth data in clinically helpful ways.

## Notes

Supplement. **eMethods.** Example of High BP Alert to Clinicians **eResults.** Comprehensive List of All Unique Potential Reasons Why No Changes Were Made to BP Care Plan, as Identified by Physician Review
