# Supplementary material for: Clinician Decisions After Notification of Elevated Blood Pressure Measurements From Patients in a Remote Monitoring Program
Source: JAMA Netw Open. 2022 Jan 14;5(1):e2143590. doi: 10.1001/jamanetworkopen.2021.43590 (PMC8760617; doi:10.1001/jamanetworkopen.2021.43590)
Supplement: Supplement. — eMethods. Example of High BP Alert to Clinicians eResults. Comprehensive List of All Unique Potential Reasons Why No Changes Were Made to BP Care Plan, as Identified by Physician Review [file jamanetwopen-e2143590-s001.pdf]

Supplementary Online Content

Lee NS, Anastos-Wallen R, Chaiyachati KH, Reitz C, Asch DA, Mehta SJ. Clinician decisions after notification of elevated blood pressure measurements from patients in a remote monitoring program. *JAMA Netw Open*. 2022;5(1):e2143590. doi:10.1001/jamanetworkopen.2021.43590

eMethods. Example of High BP Alert to Clinicians

eResults. Comprehensive List of All Unique Potential Reasons Why No Changes Were Made to BP Care Plan, as Identified by Physician Review

This supplementary material has been provided by the authors to give readers additional information about their work.

© 2022 Lee NS et al. *JAMA Network Open*.

eMethods. Example of High BP Alert to Clinicians

Ms./Mr. <last name> is participating in the SupportBP home monitoring trial for hypertension and has submitted at least 3 out of 10 elevated readings out of
control:

xxx/xxx on date
xxx/xxx on date
xxx/xxx on date

Ms./Mr. <last name> has taken his/her medication <X> out of the last 7 days, last reported on <date>.

JNC8 guidelines suggest that hypertension medications should be adjusted and added until blood pressure is controlled. Please reach out to patient to discuss.
If you have any questions regarding the SupportBP study or this message, please contact [Study Coordinator] at [XXX-XXX-XXXX].

Thank you,
<name>

eResults. Comprehensive List of All Unique Potential Reasons Why no Changes Were Made to BP Care Plan, as Identified by Physician Review

Recent medication change
Borderline values
BP values at goal
Complicated patient circumstances
Recent visit
Defer to specialist
Referral to specialist in process
Controlled in office
Spurious high reading
Downtrending/ improving values
High BP issue mixed in with another patient complaint
Alert was buried in another encounter
Multiple prior failed attempts to contact patient
Issue with BP cuff

© 2022 Lee NS et al. *JAMA Network Open*.
